# Azadirachtin Attenuates Carcinogen Benzo(a) Pyrene-Induced DNA Damage, Cell Cycle Arrest, Apoptosis, Inflammatory, Metabolic, and Oxidative Stress in HepG2 Cells

**DOI:** 10.3390/antiox12112001

**Published:** 2023-11-14

**Authors:** Annie John, Haider Raza

**Affiliations:** Department of Biochemistry and Molecular Biology, College of Medicine and Health Sciences, United Arab Emirates University, 5th Postal Region, Al Ain P.O. Box 15551, United Arab Emirates; anniej@uaeu.ac.ae

**Keywords:** HepG2 cells, Azadirachtin, benzo(a)pyrene, oxidative stress, apoptosis, DNA damage, mitochondrial dysfunction

## Abstract

Azadirachtin (AZD), a limonoid from the versatile, tropical neem tree (Azadirachta indica), is well known for its many medicinal, and pharmacological effects. Its effects as an anti-oxidant, anti-inflammatory, and anti-cancer agent are well known. However, not many studies have explored the effects of AZD on toxicities induced by benzo(a)pyrene (B(a)P), a toxic component of cigarette smoke known to cause DNA damage and cell cycle arrest, leading to different kinds of cancer. In the present study, using HepG2 cells, we investigated the protective effects of Azadirachtin (AZD) against B(a)P-induced oxidative/nitrosative and metabolic stress and mitochondrial dysfunction. Treatment with 25 µM B(a)P for 24 h demonstrated an increased production of reactive oxygen species (ROS), followed by increased lipid peroxidation and DNA damage presumably, due to the increased metabolic activation of B(a)P by CYP 450 1A1/1A2 enzymes. We also observed intrinsic and extrinsic apoptosis, alterations in glutathione-dependent redox homeostasis, cell cycle arrest, and inflammation after B(a)P treatment. Cells treated with 25 µM AZD for 24 h showed decreased oxidative stress and apoptosis, partial protection from DNA damage, and an improvement in mitochondrial functions and bioenergetics. The improvement in antioxidant status, anti-inflammatory potential, and alterations in cell cycle regulatory markers qualify AZD as a potential therapeutic in combination with anti-cancer drugs.

## 1. Introduction

Mortality due to cancer is a growing socio-economic burden globally and a cause for major concern. According to Global Cancer Statistics 2022, more than 19.3 million new cancer cases (caused by environmental influences, physiological stress, or heredity) were detected, resulting in approximately 10 million deaths in 2020 [1]. Benzo(a)pyrene (B(a)P), a polycyclic aromatic hydrocarbon (PAH), and an important toxic component of forest fires, industrial processes, vehicle exhaust, cigarette smoke, or fuel burning, can induce protein, lipid, and DNA damage, cell cycle arrest, apoptosis, inflammation, and oxidative and metabolic stress leading to different kinds of cancer [2,3]. Our earlier studies have shown increased oxidative stress, alterations in redox homeostasis, and mitochondrial dysfunction in mouse tissues exposed to cigarette smoke [4,5]. B(a)P is metabolized by detoxifying enzymes to generate by-products, which in turn form adducts with DNA, causing mutagenic and carcinogenic effects [6]. It is therefore classified as a group I carcinogen by the International Agency for Research on Cancer (IARC) [7]. B(a)P-induced toxicity has been demonstrated in various organs, including the liver, lung, and brain, causing malignant tumors [8]. Since cytochrome P450 enzymes are mainly constituted in the liver, B(a)P is mainly metabolized in the liver, making it a primary target for B(a)P toxicity [9,10]. Studies have shown an increased risk of liver cancer to environmental contact with B(a)P [11]. Dietary ingestion of B(a)P can cause hepatic steatosis due to excess ROS generation in the liver, causing cell apoptosis [12,13].

The use of natural, herbal, or dietary supplements like polyphenols has become a promising strategy as a defense against the damaging effects of B(a)P [6,14]. In vitro and in vivo studies have shown that biologically active compounds and polyphenols have the ability to reduce ROS, DNA damage, and carcinogenesis induced by B(a)P [13,14,15,16].

Numerous studies have demonstrated the effects of Azadirachtin (AZD), a limonoid extracted from the tropical neem tree (*Azadirachta indica*) as an anti-oxidant, anti-inflammatory and anti-cancer agent [17,18,19]. These researchers have shown that different parts of the neem tree, including leaves, seed kernel, seed husk, etc., have been explored to evaluate its potent anti-microbial, anti-pyretic, anti-oxidant, and other medicinal properties. Our previous studies have also shown that AZD treatment could enhance cellular survival under conditions of extreme inflammatory and oxidative stress through an interconnecting network of cell-signaling proteins [20,21]. A recent review by Nagini et al. [22] has indicated that neem limonoids including AZD demonstrate anti-carcinogenic effects in different cancer cell lines as well as in animal models by inhibiting cell proliferation, and inducing drug sensitivity, apoptosis, and anti-inflammatory responses.

Very few studies, however, have demonstrated the protection by AZD against toxicities induced by B(a)P. In vivo studies have shown Azadirachta indica leaf extract to modulate B(a)P-induced tumorigenesis in the murine forestomach and hepatic tissues [23,24,25,26]. HepG2 cells are known to possess a wide array of constitutive and inducible anti-oxidant and xenobiotic metabolizing enzymes [27,28]. Since the liver is the primary target organ for B(a)P-induced toxicities, we chose HepG2 cells as the in vitro model for our present study. In our previous studies, we reported that AZD protects pancreatic β-cells from oxidative and inflammatory stress by regulating cell proliferation and DNA damage, suppressing NF-κB signaling, and restoring redox homeostasis and mitochondrial bioenergetics [20,21]. In the present study, we aim to investigate the defense mechanism of AZD in B(a)P-induced redox and oxidative stress, DNA damage, cell cycle arrest, apoptosis, inflammation, and metabolic and mitochondrial stress, and to identify the regulatory cell signaling markers in HepG2 cells.

## 2. Materials and Methods

### 2.1. Materials

Benzo(a)pyrene [B(a)P], Azadirachtin (AZD), 3-(4,5-dimethylthiazol-2-yl)-2,5-diphenyltetrazolium bromide (MTT), propidium iodide, DTNB (5,5′-dithiobis(2-nitrobenzoic acid), reduced and oxidized glutathione (GSH/GSSG), 1-chloro 2,4-dinitrobenzene (CDNB), cumene hydroperoxide, glutathione reductase, resorufin, 7-ethoxyresorufin, methoxyresorufin, malonedialdehyde, thiobarbituric acid, apocynin, NADH, NADPH, cytochrome c, coenzyme Q2, antimycin A, dodecyl maltoside, and ATP assay kits were purchased from Sigma (St Louis, MO, USA). Kits for nitric oxide (NO) assays and mitochondrial membrane potential were purchased from R & D Systems (Minneapolis, MN, USA) and those for aconitase were purchased from Oxis Int, Inc. (Portland, OR, USA). 2,7-Dichlorofluorescein diacetate (DCFDA) and CM-H_2_XRos (reduced Mito Tracker^®^ Red) were purchased from Molecular Probes, Inc. (Eugene, OR, USA). Kits for superoxide dismutase (SOD), cleaved caspase-3, caspase-8, and caspase-9 activity assays were purchased from Abcam (Cambridge, UK) while those for catalase activity were procured from Cayman (Ann Arbor, MI, USA). Apoptosis detection kits for flow cytometry were purchased from BD Pharmingen (BD Biosciences, San Jose, CA, USA) and kits for comet assays were obtained from Cell Biolabs, Inc. (San Diego, CA, USA). HepG2 cells were obtained from the American Type Culture Collection (Manassas, VA, USA). Polyclonal antibodies against caspase-3, caspase-8, caspase-9, poly (ADP-ribose) polymerase (PARP), autophagy-related protein (Atg5), microtubule-associated light chain 3 (LC3), SQSTM1/p62, protein kinase B (AKT), phosphorylated protein kinase B (p-AKT), mammalian target of rapamycin (mTOR) and phosphorylated mammalian target of rapamycin (p-mTOR), NF–κB p65, I–κB, Bax, p53, Nrf2, and VDAC were purchased from Cell Signaling Technology, Inc. (Danvers, MA, USA), while those against CYP450 1A1 and CYP450 1A2 were from Amersham Int. Plc. (Amersham, UK). Monoclonal antibodies against cytochrome c, cyclin B1, p21, Bcl–2, Cdk2, Cdk4, and β-actin were procured from Santa Cruz Biotechnology Inc. (Santa Cruz, CA, USA), while those against SIRT-1, cyclin D1, heme oxygenase-1 (HO-1), aconitase, and histone H3 were from Abcam (Cambridge, UK). Reagents for cell culture, SDS-PAGE, and Western blot analyses were procured from Gibco BRL (Grand Island, NY, USA) and Bio-Rad Laboratories (Richmond, CA, USA).

### 2.2. Methods

#### 2.2.1. Cell Culture and Experimental Protocol

The HepG2 cell line, isolated from a liver biopsy of a 15-year-old male Caucasian was purchased from the American Type Culture Collection (Manassas, VA, USA). The cells were cultured in 100 mm cell culture petri dishes in DMEM medium in the presence of heat-inactivated fetal bovine serum (10%), non-essential amino acids (1%), 2 mM glutamine, and 100 µg/mL of penicillin/streptomycin in a 5% CO_2_–95% air incubator at 37 °C. A 10 mM stock solution of B(a)P was prepared in DMSO and diluted in the culture medium. Cells were allowed to propagate to about 60–80% confluence and then distributed into four groups: control, B(a)P-treated, AZD+B(a)P-treated and AZD-treated. Cells in the control group were treated with the vehicle alone. Cells in the B(a)P group were treated with 25 µM B(a)P for 24 h. In the AZD+B(a)P group, cells were treated with 25 µM AZD for 24 h and 25 µM B(a)P for 24 h. In the AZD group, cells were treated with 25 µM AZD alone for 24 h. The dose and time for AZD and B(a)P treatments were based on the cytotoxicity studies performed as well as our previous studies [20,21]. After treatment with AZD and/or B(a)P, the cells were trypsinized, washed and homogenized in a cold H-medium buffer, pH 7.4, containing 70 mM sucrose, 220 mM mannitol, 2.5 mM HEPES, 2 mM EDTA, and 0.1 mM phenylmethylsulfonyl fluoride. Nuclear, mitochondrial, and post-mitochondrial fractions were isolated via sub-cellular fractionation and protein concentrations were measured as described previously [20,21,29].

#### 2.2.2. Cytotoxicity and Apoptosis Measurement

A MTT assay was used to determine the mitochondrial dehydrogenase-based cellular viability. In short, cells were grown in 96-well plates and treated with different concentrations (0–100 µM) of B(a)P or AZD for 24 h–48 h. Cell viability was assessed via the formation of insoluble purple formazan crystals, caused by the reduction of MTT dye, which were then dissolved in acidified alcohol and measured using an ELISA reader (TECAN Infinite M200 PRO, Austria) at 550 nm as described before [20,29].

Cell apoptosis was measured in HepG2 cells treated with B(a)P and/or AZD via flow cytometry according to the vendor’s protocol (BD Pharmingen, BD Biosciences, San Jose, USA) as described before [20,29]. In brief, HepG2 cells treated with B(a)P and/or AZD for 24 h were harvested, and re-suspended in the recommended binding buffer after washing with PBS. A fraction of the cell suspension (100 µL/containing 1 × 10^5^ cells) was then treated for 15 min with a combination of Annexin V conjugated to FITC and propidium iodide (PI) at room temperature. A binding buffer was then added to resuspend the cell suspension and apoptosis was measured immediately using a Becton Dickinson FACSCanto II analyzer. With this protocol, the viable, apoptotic, and necrotic/dead cells could be distinguished.

Activities of the different caspases, namely, 3, 8 and, 9 were measured in the HepG2 cells treated with B(a)P and/or AZD per the vendor’s instructions. In brief, the cell lysates from control HepG2 and AZD and/or B(a)P-treated cells were incubated with p-nitro aniline-conjugated caspase-specific peptide substrates, DEVD IETD, and LEHD, respectively. The cleavage of the peptide by the respective caspases resulted in the release of the chromophore, which was measured colorimetrically at a wavelength of 405 nm as described before [20,21,29].

#### 2.2.3. DNA Damage and Cell Cycle Analysis

DNA damage was assessed using the DNA laddering assay by loading the samples on 2% agarose gels and staining the fragments with ethidium bromide after electrophoresis as described earlier [20,29]. Cellular DNA damage was confirmed using the comet assay. Per the manufacturer’s instructions, the cells were washed and centrifuged after treatment. Cells were resuspended in cold PBS at a concentration of 1 × 10^5^ cells/ml and low-melting-point agarose was added in a 1:10 ratio, mixed, and transferred to 3-well glass slides. The slides were kept at 4 °C for 15 min and then incubated for 1 h at 4 °C in lysis buffer followed by incubation in NaOH solution (1.2%) for a further 30 min. The slides were then run in a horizontal electrophoresis chamber at 1volt/cm containing 300 mM NaOH and 1 mM EDTA, pH > 13, as an electrophoresis buffer, stained with Vista Green DNA dye at room temperature for 15 min and analyzed using an Olympus fluorescence microscope as described before [30].

Flow cytometry was used to assess the distribution of cells in the different phases of the cell cycle after B(a)P and/or AZD treatment for 24 h, which was assayed by measuring the DNA content of propidium iodide (PI)-labeled nuclei as described before [20]. Briefly, control and B(a)P/AZD-treated cells were fixed with 70% ethanol overnight at −20 °C after resuspending the cells in cold PBS. Cells were then washed with cold PBS, treated with 0.15% RNase A and 80 µg/ml of PI at 37 °C for 30 min. Total DNA content was measured at an excitation wavelength of 488 nm with detection at 620 nm using FACSCanto II Flow Cytometer (Beckton Dickinson, San Jose, CA, USA). Results were expressed as %age DNA distribution in each phase.

#### 2.2.4. Measurement of Reactive Oxygen Species (ROS)

A cell-permeable probe, DCFDA, was used to measure ROS production in the different sub-cellular fractions. Briefly, control and B(a)P- and/or AZD-treated cells were incubated with 5 µM DCFDA for 30 min at 37 °C and washed with PBS, and the fluorescence read at an excitation wavelength of 488 nm and an emission wavelength of 525 nm using the ELISA reader (TECAN Infinite M 200 PRO, Grödig, Austria). Apocynin-sensitive plasma membrane-bound ROS as well as mitochondrial ROS were measured as described before [20,31]. Cells grown on coverslips were also treated and incubated with DCFDA as described above and the fluorescence was measured microscopically as described before [20,31]. The fluorescence caused by ROS production was also measured via flow cytometry [20,31].

To complement intracellular ROS production, we evaluated the production of mitochondrial ROS by ROS-specific staining using the dye CM-H_2_XRos (reduced Mito Tracker^®^ Red). Briefly, control and B(a)P- and/or AZD-treated HepG2 cells, seeded on coverslips, were treated with freshly prepared CM-H_2_XRos and incubated at room temperature for 15 min. After washing and fixing with 3.7% formaldehyde, the coverslips were mounted on slides and visualized using the Olympus fluorescence microscope.

#### 2.2.5. Measurement of Lipid Peroxidation (LPO), Nitric Oxide (NO) Levels, and SOD/Catalase Activities

Lipid peroxidation was measured using malonedialdehyde as a standard via the thiobarbituric acid method as described before [5,32]. The total nitrite in the culture supernatants was measured as an indicator of NO levels in the control and treated cells using the Griess reagent per the vendor’s protocol [21,31].

The activities of anti-oxidant enzymes, SOD, and catalase were measured using the respective kits per the manufacturer’s instructions. The activity of xanthine oxidase to convert xanthine into uric acid and hydrogen peroxide, resulting in the reduction of NBT (nitro blue tetrazolium) to NBT-formazan, was a measure of SOD, which was measured colorimetrically at 550 nm. This reduction of NBT caused by superoxide anions was inhibited by SOD, which was used to measure the SOD activity in the control and treated samples.

Catalase activity was measured based on its ability to catalyze the oxidation of methanol, and the formaldehyde thus formed in the presence of a chromogen was read colorimetrically at 450 nm.

#### 2.2.6. Measurement of GSH Metabolism and Redox Homeostasis

Total glutathione (GSH) levels and its metabolism and redox homeostasis were measured in the mitochondrial and post-mitochondrial fractions of the control and B(a)P- and/or AZD-treated cells. Total GSH levels was a measure of the reduction of oxidized to reduced glutathione by glutathione reductase using DTNB (5,5′-dithiobis (2-nitrobenzoic acid) as a substrate. The activities of glutathione S-transferase (GST) using CDNB as a substrate, glutathione reductase (GSH-reductase) using GSSG/NADPH as a substrate and glutathione peroxidase (GSH-Px) using cumene hydroperoxide as a substrate were measured, using standard protocols as described before [21,30,32].

#### 2.2.7. Measurement of CYP 450 Activities

Post-mitochondrial fractions from control and B(a)P- and/or AZD-treated cells were analyzed for CYP 450 1A1 and 1A2 activities using 7-ethoxyresorufin and methoxy resorufin, respectively, as substrates as described before [30].

#### 2.2.8. Measurement of Mitochondrial Membrane Potential (MMP)

Mitochondrial membrane potential (MMP) was measured in control HepG2 cells or treated with B(a)P and/or AZD for 24 h using DePsipher ^TM^ (R & D Systems, Minneapolis, MN, USA), a fluorescent cationic dye. The dye remains in its green fluorescent form when membrane potential is reduced. This was measured using a Becton Dickinson FACSCanto II analyzer as described before [21,29].

#### 2.2.9. Measurement of Bioenergetics and Activities of Mitochondrial Enzymes

Respiratory enzyme complexes were measured in the presence of lauryl maltoside, in the mitochondrial fractions of control or B(a)P- and/or AZD-treated HepG2 cells. The activities of mitochondrial respiratory Complex I (NADH-ubiquinone oxidoreductase) was measured using coenzyme Q_2_ as a substrate, those of Complex II/III (succinate-ubiquinone oxidoreductase/ubiquinol-cytochrome c oxidoreductase) were measured using succinate/cytochrome c as a substrate, and those of Complex IV (cytochrome c oxidase) were measured using reduced cytochrome c as a substrate in accordance with the method of Birch-Machin and Turnbull [33].

The ATP content in the control and B(a)P- and/or AZD-treated cells was measured using an ATP Bioluminescent cell assay kit per the vendor’s protocol, and luminescence was measured using TD-20/20 Luminometer (Turner Designs, Sunnyvale, CA, USA).

Aconitase activity was measured using the aconitase assay kit (Oxis Int, Inc., Portland, OR, USA) per the manufacturer’s instructions.

#### 2.2.10. SDS-PAGE and Western Blot Analysis

Proteins (5–25 µg) from the different cellular fractions from control and B(a)P- and/or AZD-treated HepG2 cells were loaded on 12% sodium dodecyl sulfate polyacrylamide gels and resolved via electrophoresis. At the end of the run, the resolved proteins from the gel were electrophoretically transferred onto a nitrocellulose membrane via Western blotting as described before [21,29]. The blots containing the transferred proteins were then blocked using 5% non-fat milk in Tris-buffered saline containing Tween-20 and then probed with primary antibodies against cytochrome c (1:1000), Bax (1:1000), Bcl-2 (1:200), aconitase (1:500), NF-κB p65 (1:1000), I-κB (1:1000), SIRT-1 (1:8000), CYP 450 1A1(1:500) and 1A2 (1:500), PARP (1:1000), caspase-8 (1:1000), caspase-9 (1:1000), cleaved caspase-3 (1:500), Nrf2 (1:500), HO-1 (1:250), Atg-5 (1:1000), LC3 (1:1000), p62 (1:1000), Akt (1:1000), p-Akt (1:2000), mTOR (1:2000), p-mTOR (1:2000), p53 (1:200), p21 (1:100), cyclin B1 (1:1000), cyclin D1 (1:10,000), Cdk2 (1:200), and Cdk4 (1:200) overnight at 4 °C. After incubation with the primary antibodies, the blots were washed and then incubated with the required secondary antibodies, goat anti-rabbit (1:2000) or goat anti-mouse (1:20,000), for 1 h at room temperature. After washing, the immunoreactive proteins were visualized via enhanced chemiluminescence using the Sapphire Biomolecular Imager (Azure biosystems, Dublin, CA, USA) or by developing them on X-ray films. Beta-actin (1:200), VDAC (1:1000), and histone H3 (1:1000) were used as loading controls for total/post-mitochondrial, mitochondrial and, nuclear fractions, respectively. Quantitative band intensity was measured using Image Studio Lite Ver.5.2 (LI-COR Biosciences, Lincoln, NE, USA) and expressed as relative ratios normalized to their appropriate loading proteins.

#### 2.2.11. Statistical Analysis

Values represent the mean ± SD of three individual replicated assay results. SPSS software (version 23) was used to assess the statistical significance of the data, via an analysis of variance (ANOVA) followed by LSD post hoc analysis. *p*-values of <0.05 were considered statistically significant.

## 3. Results

### 3.1. Differential Effects of LPS and AZD on Cell Viability, and Apoptosis

**Figure 1:**
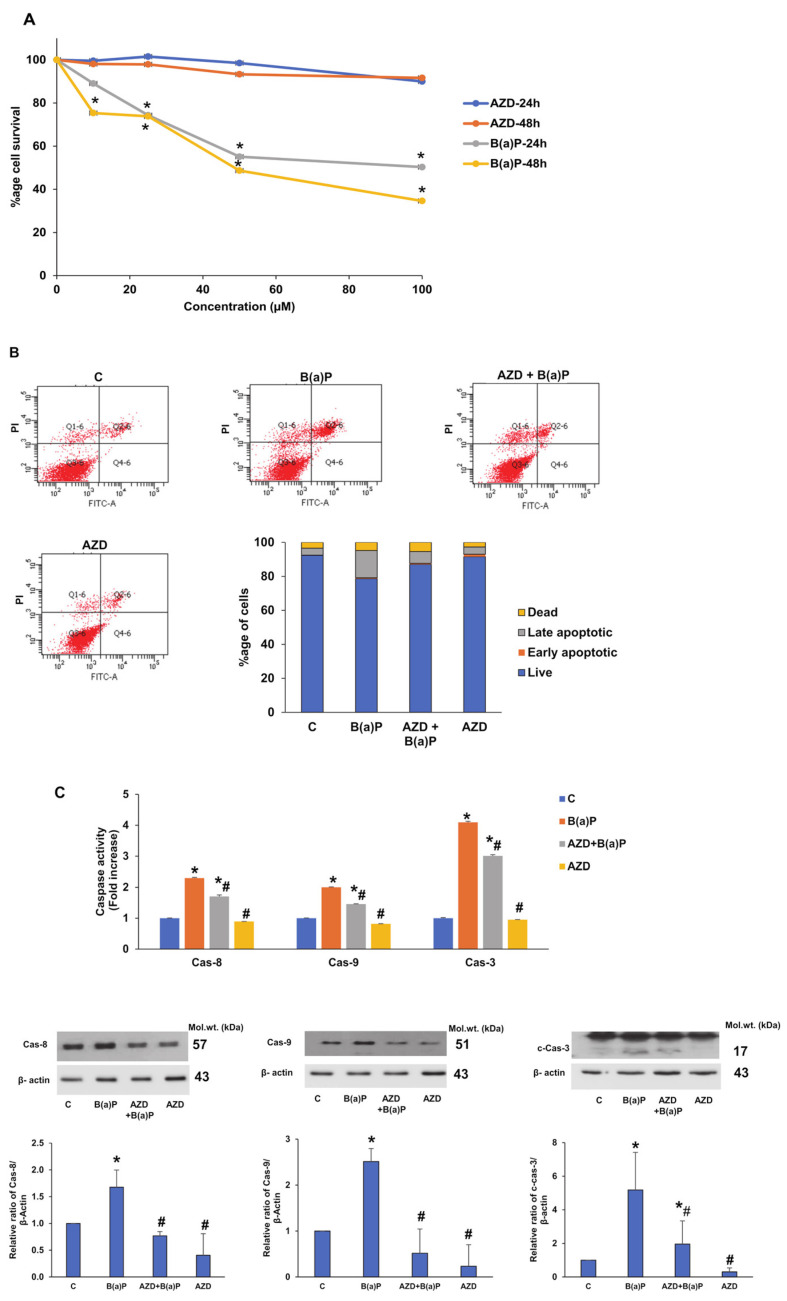
B(a)P-induced effects on cell survival and apoptosis, and protection by AZD in HepG2 cells. After the treatment of HepG2 cells with different doses of B(a)P or AZD (10 µM to 100 µM) for 24 h and 48 h, cell survival was evaluated using the MTT assay (**A**). HepG2 cells treated with 25 µM B(a)P for 24 h and/or 25 µM AZD for 24 h, and apoptosis measured via flow cytometry (**B**). A typical flow cytometric dot plot showing the quantitation of cells in each of the quadrants from three replicated experiments, and the average percentage values plotted as a stacked histogram. The chromophore released by the activities of caspases, mainly caspases-3, -8, and -9, were measured colorimetrically in cells treated with 25 µM B(a)P and/or 25 µM AZD for 24 h, using their respective substrates at 405 nm (**C**). The expression of the caspase enzymes was confirmed via immunoblotting. Values indicate the mean ± SD of three replicate experiments. Asterisks represent significant differences and are fixed at *p* ≤ 0.05 (* represents significant differences with respect to control cells whereas # represents significant differences with respect to B(a)P-treated cells). Molecular weights are represented in kDa.

HepG2 cells were treated with different concentrations (10 µM, 25 µM, 50 µM, and 100 µM) of AZD or B(a)P alone at 24 h and 48 h, and cell survival was assessed using the mitochondrial dehydrogenase-based MTT assay. Figure 1A shows the cell survival after the treatment of HepG2 cells with either B(a)P or AZD alone. No significant changes were observed with the different doses of AZD at different time intervals. However, a decrease in cell survival (30–50%) was observed in the treatment of cells with 25 µM–100 µM B(a)P for 24 h, which decreased still further (30–70%) at 48 h. Based on these results and a literature survey, we used 25 µM B(a)P for 24 h for our further study. Also, based on our previous studies [20,21], we used 25 µM AZD for 24 h alone or in combination with B(a)P to study the effects of B(a)P-induced toxicity on HepG2 cells.

Foremost, we investigated the effects of B(a)P alone or in combination with AZD on apoptosis in the HepG2 cells (Figure 1B). We observed around 16% of cells in the late apoptotic stage after treatment with 25 µM B(a)P for 24 h. AZD treatment alone or in the presence of B(a)P showed around 4–7% cells in late apoptosis, suggesting the protection of HepG2 cells from the cytotoxicity of B(a)P.

To further verify the apoptotic effects of B(a)P, we studied the activities and expression of the caspase enzymes (Figure 1C) known to play an important role in programmed cell death (apoptosis). We observed a significant two- to three-fold increase in the activities as well as expression of caspases-8, -9, and -3 enzymes. This indicated that both the extrinsic as well as intrinsic pathways for apoptosis were activated after B(a)P treatment. As observed earlier, AZD treatment alone or in the presence of B(a)P significantly reduced the activities as well as expression of the enzymes, which again indicates the protection of HepG2 cells against B(a)P-induced toxicity by AZD.

### 3.2. Attenuation of B(a)P-Induced DNA Damage and Cell Cycle Perturbation by AZD

B(a)P is known to undergo metabolic activation to form diol-epoxides, which form adducts with DNA, causing DNA damage, errors in DNA replication, and disturbances in the cell cycle. We therefore tried to assess the protection of AZD on DNA break-down and cell cycle arrest caused by B(a)P.

**Figure 2:**
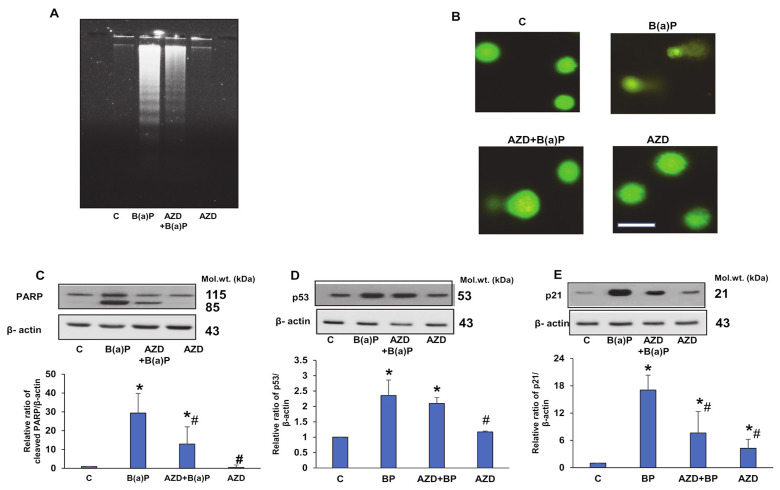
DNA breakdown and activation of DNA repair enzymes caused by B(a)P in HepG2 cells. To asses for DNA damage in HepG2 cells treated with 25 µM B(a)P and/or 25 µM AZD for 24 h, DNA samples were run on 2% agarose gels and stained with ethidium bromide (**A**). DNA injury was also visualized using the single-cell gel electrophoresis assay in accordance with the manufacturer’s recommendation (**B**). Scale bar indicates 50 µm. Comet tails indicate cells showing damaged DNA. Representative results from control and B(a)P alone and/or AZD from three replicate experiments are shown. Expression of the DNA repair enzymes PARP (**C**), p53 (**D**), and its downstream protein, p21 (**E**) were checked via immunoblotting. β-actin was used as the loading control. Values indicate the mean ± SD of three replicate experiments. Asterisks represent significant differences and are fixed at *p* ≤ 0.05 (* represents significant differences with respect to control cells whereas # represents significant differences with respect to B(a)P-treated cells). Molecular weights are represented in kDa.

As shown in Figure 2A, DNA laddering was observed via agarose gel electrophoresis on the treatment of HepG2 cells with B(a)P, which was attenuated in the presence of AZD. No laddering was observed with AZD alone. To further confirm the DNA damage by B(a)P, we performed a comet assay or single-cell gel electrophoresis assay. Figure 2B shows augmented DNA breakdown by B(a)P, which was attenuated in the presence of AZD.

We then checked the expression of PARP, a DNA repair enzyme in HepG2 cells, after treatment with B(a)P and/or AZD. As shown in Figure 2C, B(a)P treatment caused PARP cleavage, which again confirms DNA breakdown. The cleavage was significantly reduced in the presence of AZD. DNA damage is also known to induce the activation of p53, which helps in DNA repair by inducing cell cycle arrest, giving cells time to repair the damaged DNA. In our study, we also observed an increased activation of p53 (by almost two-fold), which reduced slightly upon treatment with AZD (Figure 2D). In order to induce cell cycle arrest, p53 activates the downstream p21 protein, an inhibitor of Cdks (cyclin-dependent kinases), required for cell cycle progression. Our study also demonstrated an almost two-fold increase in the level of p21 protein, which again reduced significantly in the presence of AZD (Figure 2E).

**Figure 3:**
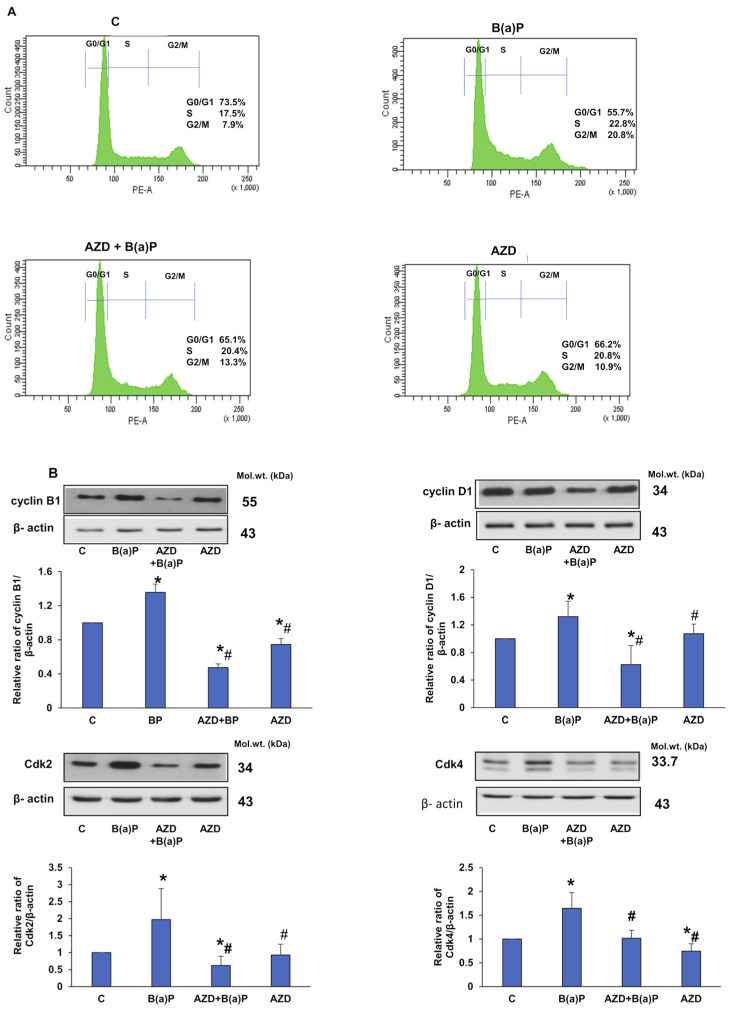
B(a)P-induced perturbations in the cell cycle and its regulatory markers in HepG2 cells treated with B(a)P and/or AZD. To assess cell cycle distribution, cells were stained with propidium iodide and the fluorescence was quantitated using FACSCanto II Flow Cytometer at an excitation wavelength of 488 nm with detection at 620 nm. DNA distribution in each phase from three replicate experiments was quantitated. A typical histogram showing the % of DNA distribution in the individual phases is shown in (**A**). The expression of the cell cycle regulatory markers, cyclins B1 and D1, and Cdks 2 and 4 is shown in (**B**). β-actin was used as the loading control. Blots were densitometrically quantitated and are represented as the mean ± SD of three replicate experiments. Asterisks represent significant differences and are fixed at *p* ≤ 0.05 (* represents significant differences with respect to control cells whereas # represents significant differences with respect to B(a)P-treated cells). Molecular weights are represented in kDa.

We further investigated the effects of B(a)P and/or AZD on cell cycle distribution and cell cycle regulatory proteins. Figure 3A shows a significant increase in cell distribution in the S and G2/M phases and a concomitant reduction in the G0/G1 phase after the treatment of HepG2 cells with 25 µM B(a)P for 24 h. Treatment with AZD showed a significant reduction in cells in the G2/M phase and an increase in cell percentage in the G0/G1 phase. To further elucidate the mechanism underlying these cell cycle perturbations, we investigated the effects of B(a)P and/or AZD on cell cycle regulatory proteins. We observed increased expressions of cyclin B1, cyclin D1, Cdk2, and Cdk4 with B(a)P treatment, which were attenuated significantly in the presence of AZD (Figure 3B).

### 3.3. Effects of B(a)P and/or AZD on Oxidative Stress and Redox Homeostasis

**Figure 4:**
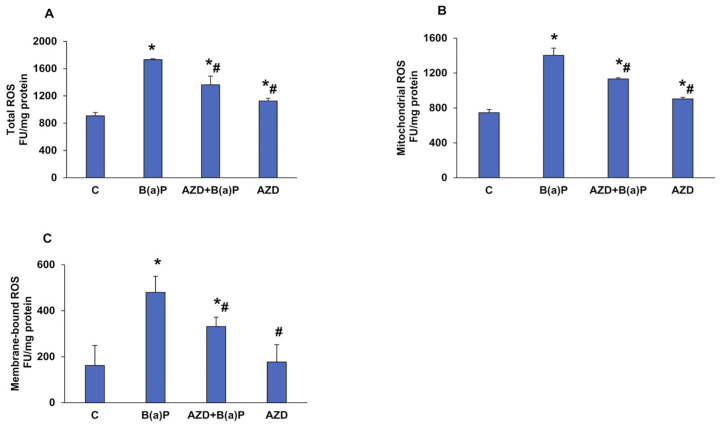

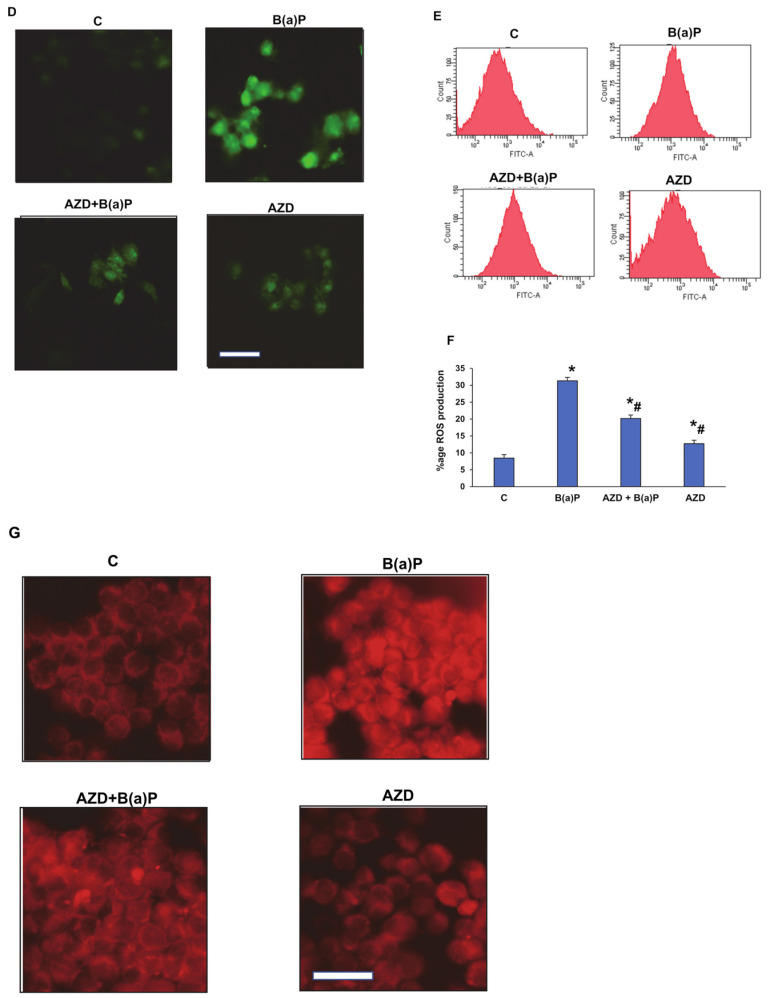
B(a)-induced ROS production in HepG2 cells. Intracellular production of ROS in the different sub-cellular fractions was measured using DCFDA, a cell-permeable fluorescent probe, in Hep G2 cells treated with B(a)P and/or AZD for 24 h using the ELISA reader (TECAN Infinite M 200 PRO, Austria). Total ROS production (**A**), membrane-bound ROS (**B**), and mitochondrial ROS (**C**) as measured. Cells grown on coverslips treated and incubated with DCFDA as above and the fluorescence was measured microscopically (**D**). The fluorescence caused by ROS production measured via flow cytometry (**E**). The % of ROS production calculated and shown as a histogram (**F**). The production of mitochondrial ROS, confirmed using a mitochondrial ROS-specific dye, CM-H_2_XRos (reduced Mito Tracker^®^ Red), and visualized using the Olympus fluorescence microscope (**G**). The scale bar in (**D**,**G**) represents 50 µm. Values are represented as the mean ± SD of three replicate experiments. Asterisks represent significant differences and are fixed at *p* ≤ 0.05 (* represents significant differences with respect to control cells whereas # represents significant differences with respect to B(a)P-treated cells).

Previous studies have reported B(a)P-induced oxidative stress in vitro as well as in vivo [3,6,13,14]. We also observed a two-fold increase in total ROS and mitochondrial ROS production as well as membrane-bound NADPH oxidase-dependent ROS production (Figure 4A–C) after B(a)P treatment, using the DCFDA fluorescent probe. Treatment with AZD significantly reduced ROS production in all the three sub-cellular fractions. Total ROS production was also confirmed via microscopy (Figure 4D) and flow cytometry (Figure 4E).

To further confirm mitochondrial ROS production due to B(a)P-induced oxidative stress, we microscopically analyzed HepG2 cells untreated or treated with B(a)P and/or AZD with a ROS-sensitive, mitochondrial-specific fluorogenic probe, CM-H2XRos (Mito Tracker^®^Red). The increased red stain in cells treated with B(a)P indicates the entry and accumulation of this dye in the mitochondria due to oxidation by intracellular ROS (Figure 4F).

**Figure 5:**
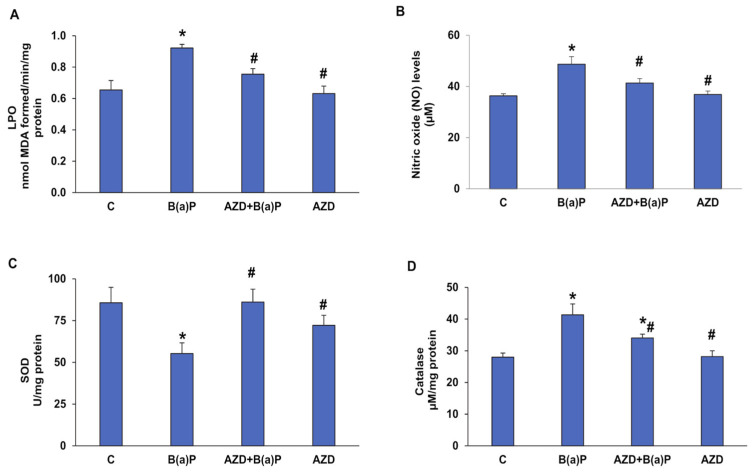
B(a)-induced oxidative stress in HepG2 cells. NADPH-dependent lipid peroxidation in HepG2 cells treated with B(a)P and/or AZD, measured as total malonedialdehyde produced (**A**). The total nitrite in the culture supernatants was determined using Griess reagent as a measure of NO levels (**B**). SOD activity determined via the reduction of NBT (nitroblue tetrazolium) to NBT-formazan per the vendor’s instructions (**C**) and catalase activity as a measure of the formaldehyde formed (**D**). Values are represented as mean ± SD of three replicate experiments. Asterisks represent significant differences and are fixed at *p* ≤ 0.05 (* represent significant differences with respect to control cells whereas # represent significant differences with respect to B(a)P-treated cells).

To further decipher the prospective role of B(a)P-induced oxidative/nitrosative stress, we measured the levels of malonedialdehyde (MDA) and nitric oxide (NO) and also the levels of anti-oxidant enzymes, SOD, and catalase in HepG2 cells treated with B(a)P and/or AZD for 24 h. We observed a 40% increase in lipid peroxidation (Figure 5A) and a 34% increase in NO levels (Figure 5B) compared to those in control HepG2 cells after treatment with 25 µM B(a)P for 24 h. A decrease of about 20% in both oxidant levels was observed after treatment with 25 µM AZD for 24 h.

Similarly, alterations were also observed in the activities of oxidative stress-sensitive anti-oxidant enzymes, SOD, and catalase. A decrease of around 40% in SOD activity (Figure 5C) and almost 47% increase in catalase activity (Figure 5D) compared to control cells were observed after B(a)P treatment. AZD brought the activities of SOD and catalase close to control levels, reducing the imbalance between oxidant and anti-oxidant levels and thus reducing oxidative stress.

**Figure 6:**
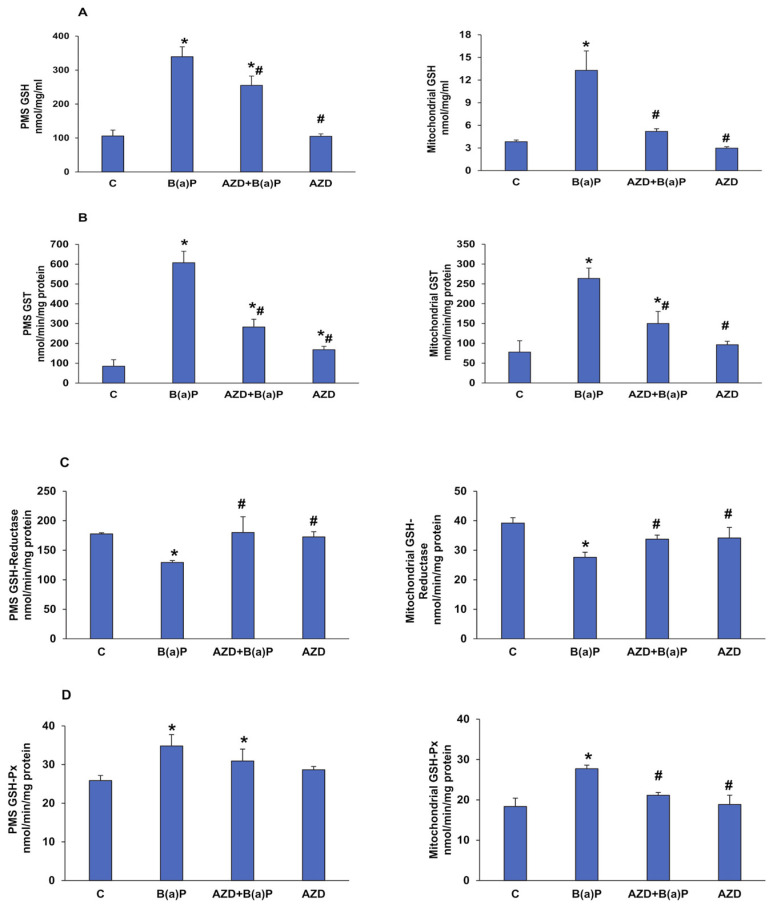
Alterations in redox homeostasis in the sub-cellular fractions of B(a)P- and/or AZD- treated HepG2 cells. Total glutathione (GSH) levels measured in the mitochondrial and post-mitochondrial fractions of the control and B(a)P and/or AZD-treated cells as a measure of the conversion of oxidized glutathione into reduced glutathione using DTNB (5,5′-dithiobis (2-nitrobenzoic acid) as a substrate (**A**). CDNB, GSSG/NADPH, and cumene hydroperoxide used as substrates to measure the activities of glutathione S-transferase (GST) (**B**), glutathione reductase (GSH-reductase) (**C**). and glutathione peroxidase (GSH-Px) (**D**), respectively, in the mitochondrial and post-mitochondrial fractions of the control and B(a)P- and/or AZD-treated cells. Values are represented as the mean ± SD of three replicate experiments. Asterisks represent significant differences and are fixed at *p* ≤ 0.05 (* represents significant differences with respect to control cells whereas # represents significant differences with respect to B(a)P-treated cells).

We further checked the GSH-dependent redox homeostasis in these cells after treatment with B(a)P and/or AZD for 24 h. A marked increase (of almost three-fold) in the levels of total GSH (Figure 6A) and the activity of the GSH-metabolizing enzyme, glutathione S-transferase (GST), was observed in the mitochondrial as well as the post-mitochondrial (PMS) fractions of HepG2 cells treated with 25 µM B(a)P for 24 h (Figure 6B). Previous studies have reported the overproduction of GSH in 24 h after B(a)P treatment as an endogenous mechanism to conjugate with and facilitate the detoxification of B(a)P and its metabolites, catalyzed by GSTs [28,34].

Further, we also observed a decrease (about 30%) in the activity of glutathione reductase (GSH-reductase) (Figure 6C) and a 30–50% increase in glutathione peroxidase (GSH-Px) activity (Figure 6D) after B(a)P treatment compared to that of the control untreated cells in the sub-cellular fractions. These alterations could be due to the increased ROS such as the H_2_O_2_ produced during the metabolism of the toxic metabolites and the recycling of oxidized GSSG produced in the reaction. These alterations were partially protected by the AZD treatment.

### 3.4. Effects of B(a)P and/or AZD on Phase I Drug-Metabolizing Enzymes

**Figure 7:**
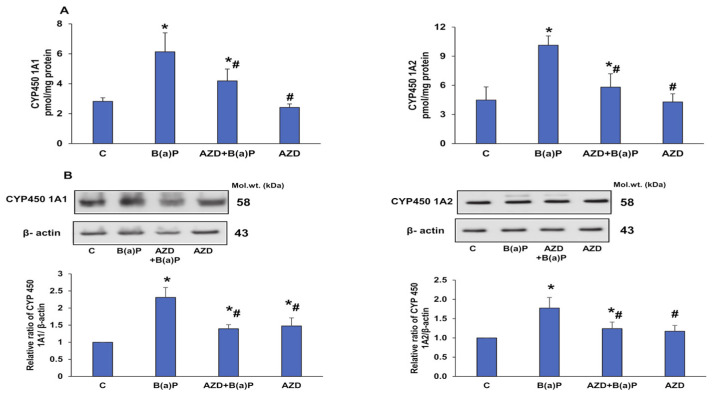
Effects of B(a)P and/or AZD on CYP 450 activities in HepG2 cells. CYP 450 1A1 and 1A2 activities measured in the post-mitochondrial fractions from control and B(a)P and/or AZD-treated cells using 7-ethoxyresorufin and methoxy resorufin as substrates, respectively (**A**). The protein expression of CYP 450 1A1 and 1A2 (**B**). β-actin was used as the loading control. Activity measurement histograms and a quantitation of the blots are represented as the mean ± SD of three replicate experiments. Asterisks represent significant differences and are fixed at *p* ≤ 0.05 (* represents significant differences with respect to control cells whereas # represents significant differences with respect to B(a)P-treated cells). Molecular weights are expressed in kDa.

It is a well-known fact that B(a)P metabolism involves the CYP 450 1A and the GST detoxifying enzymes [28]. Our study also demonstrated a two-fold induction of the activities of CYP 450 1A1 and 1A2 enzymes (Figure 7A) after treatment with B(a)P. This was further confirmed by the increased expression of these enzymes upon Western blotting (Figure 7B).

### 3.5. Effects of B(a)P and/or AZD on Mitochondrial Functions and Bioenergetics

**Figure 8:**
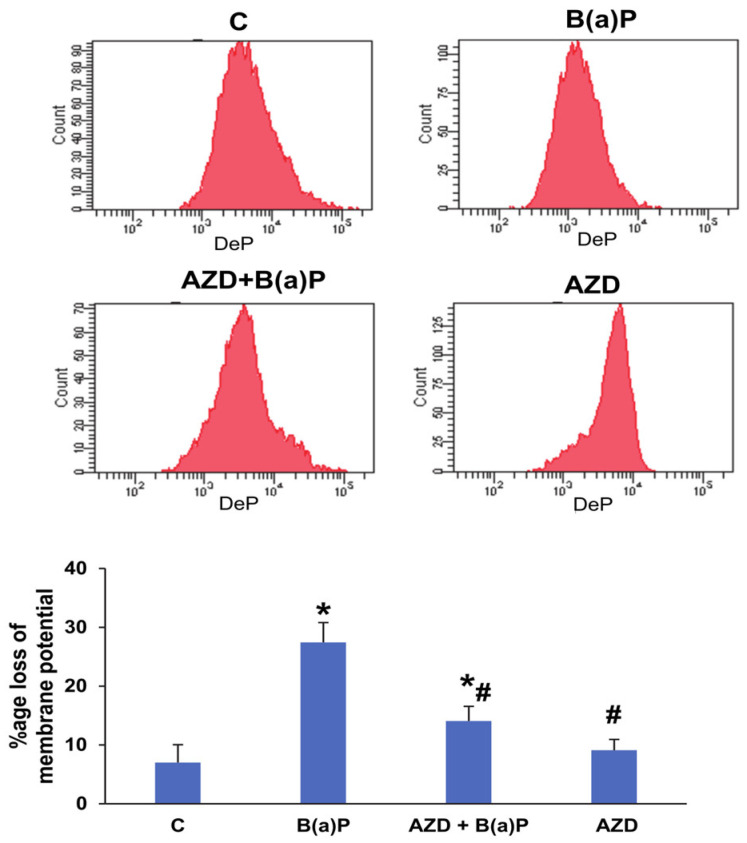
B(a)P-induced alterations in mitochondrial membrane potential (MMP) in HepG2 cells. MMP was measured in HepG2 cells after treatment with B(a)P and/or AZD via flow cytometry using DePsipher ^TM^ (R & D Systems, Minneapolis, MN, USA), a fluorescent cationic dye. The bar diagram shows the loss of membrane potential (%age) and the data are expressed as the mean ± SD of three replicate experiments. Asterisks represent significant differences and are fixed at *p* ≤ 0.05 (* represents significant differences with respect to control cells whereas # represents significant differences with respect to B(a)P-treated cells).

Our study showed increased ROS production in the mitochondria after B(a)P treatment in HepG2 cells. In continuation, we checked for perturbations in mitochondrial membrane potential and the activities of respiratory complexes. We observed a significant 3-fold loss of membrane potential in HepG2 cells treated with B(a)P while AZD showed partial protection (Figure 8).

**Figure 9:**
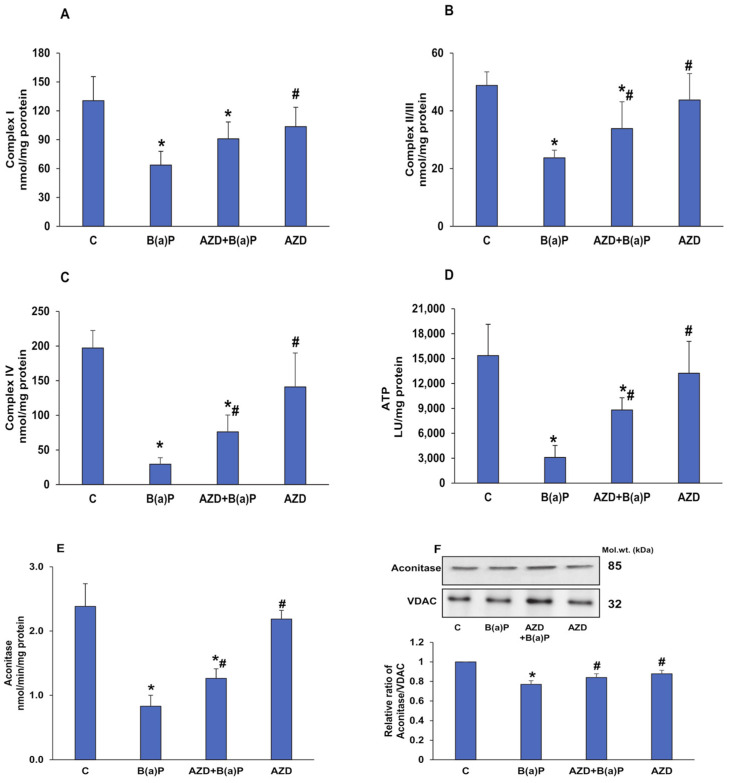
B(a)P-induced alterations in the activities of the respiratory enzyme complexes and bioenergetics. Mitochondrial respiratory complex activities, Complex I (**A**), Complex II/III (**B**), and Complex IV (**C**) measured in HepG2 cells treated with B(a)P and/or AZD, using their respective substrates. ATP production (**D**) measured using the Bioluminescent assay kit per the vendor’s instructions. The activity of Aconitase (**E**), a ROS-sensitive Krebs’s cycle enzyme, measured using the Aconitase assay kit and the expression of the enzyme (**F**) checked via immunoblotting and densitometric analysis. Activity measurement histograms and a quantitation of the blots are represented as the mean ± SD of three replicate experiments. Asterisks represent significant differences and are fixed at *p* ≤ 0.05 (* represents significant differences with respect to control cells whereas # represents significant differences with respect to B(a)P-treated cells). Molecular weights are expressed in kDa.

B(a)P caused an almost 50% inhibition of the activities of mitochondrial respiratory Complexes I and II/III, and an 80% inhibition of Complex IV activity (Figure 9A–C). This was again partially protected by AZD. In support of this, a significant reduction (almost 80%) in ATP levels was also observed (Figure 9D). Again, partial recovery was observed after AZD treatment. AZD alone, however, did not cause much of a difference in activities compared to those in the control cells.

Almost 70% inhibition was also observed in the activity of the Krebs’ cycle enzyme, aconitase (Figure 9E), after B(a)P treatment, with slight recovery with AZD. The decreased expression of this ROS-sensitive mitochondrial matrix enzyme further confirmed this finding (Figure 9F).

**Figure 10:**
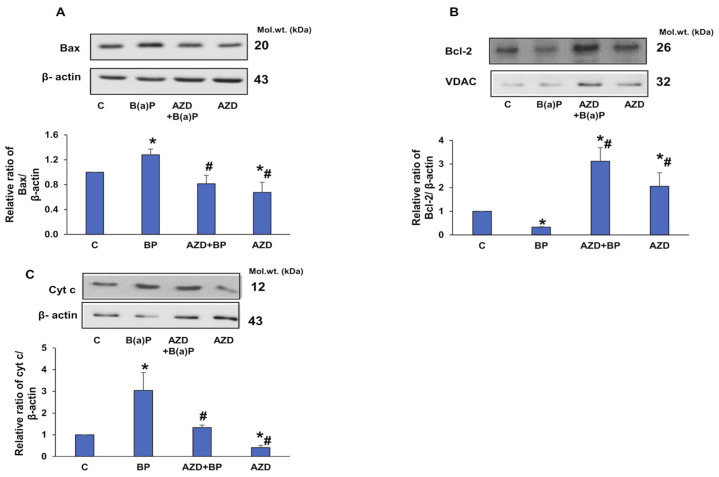
Expression of mitochondrial oxidative stress markers induced by B(a)P. Sub-cellular fractions (mitochondria and post-mitochondria) from HepG2 cells treated with B(a)P and/or AZD resolved using 12% SDS-PAGE, transferred via Western blotting, and immunoblotted using the protein-specific antibodies against Bax (**A**), Bcl-2 (**B**), and cytochrome c (**C**). Immunoreactive proteins were visualized via enhanced chemiluminescence using Sapphire Biomolecular Imager (Azure biosystems, Dublin, OH, USA) or by developing them on X-ray films. Beta-actin and VDAC were used as loading controls for total/post-mitochondrial and mitochondrial fractions, respectively. Image Studio Lite Ver.5.2 (LI-COR Biosciences, Lincoln, NE, USA) software was used for the densitometric analysis of the protein bands and these were plotted as ratios relative to their appropriate loading proteins and are represented as histograms. A typical representation of at least three replicate experiments is shown. Asterisks represent significant differences and are fixed at *p* ≤ 0.05 (* represents significant differences with respect to control cells whereas # represents significant differences with respect to B(a)P-treated cells). Molecular weights are expressed in kDa.

In addition, the increased expression of Bax and reduced expression of Bcl-2 with release of cytochrome c into the cytosol are indicators of mitochondrial oxidative stress after B(a)P treatment (Figure 10A–C). This could have triggered the mitochondrial apoptotic cascade, resulting in the activation of caspase-9 and caspase-3 (as shown in Figure 1C). The recovery of the anti-apoptotic proteins with a decrease in the pro-apoptotic proteins was seen after AZD treatment, indicating protection from B(a)P-mediated toxicity.

### 3.6. B(a)P-Induced Expression of Inflammatory, Anti-Oxidant, and Autophagy Markers

**Figure 11:**
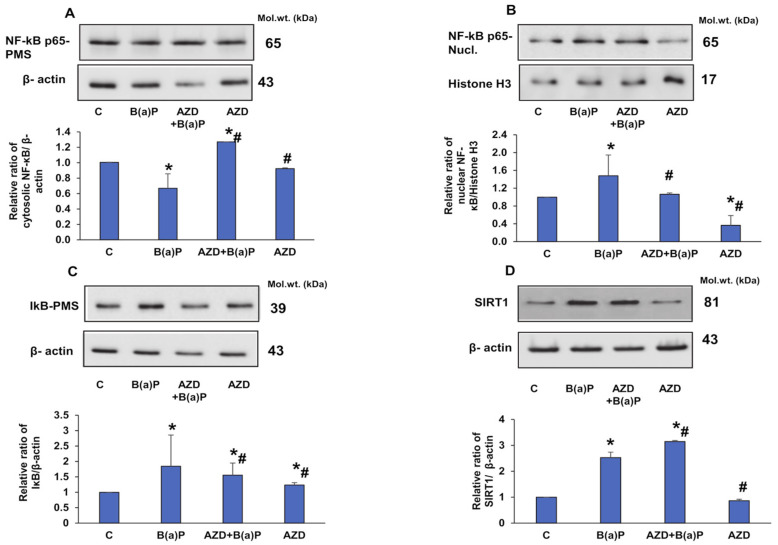
Expression of inflammatory stress markers induced by B(a)P. Sub-cellular fractions (nuclear and post-mitochondria) from HepG2 cells treated with B(a)P and/or AZD resolved using 12% SDS-PAGE, transferred via Western blotting, and immunoblotted with antibodies against NF-κB p65 cytosolic (**A**), NF-κB p65 nuclear (**B**), IκB (**C**), and SIRT 1 (**D**). Immunoreactive proteins were visualized via enhanced chemiluminescence using Sapphire Biomolecular Imager (Azure biosystems, Dublin, CA, USA) or by developing them on X-ray films. Beta-actin and Histone H3 were used as loading controls for total/post-mitochondrial and nuclear fractions, respectively. Image Studio Lite Ver.5.2 (LI-COR Biosciences, Lincoln, NE, USA) was used for densitometric analysis and the band intensities are represented as ratios of the specific proteins relative to their appropriate loading proteins and as histograms. A typical representation of three replicate experiments is shown. Asterisks represent significant differences and are fixed at *p* ≤ 0.05 (* represents significant differences with respect to control cells whereas # represents significant differences with respect to B(a)P-treated cells). Molecular weights are expressed in kDa.

Increased translocation of the inflammatory marker NF-κB p65 into the nucleus with an increased expression of the inhibitory I-κB protein in the cytosol was observed after B(a)P treatment (Figure 11A–C). AZD treatment decreased the expression of I-κB protein and decreased the translocation of NF-κB p65, indicating the anti-inflammatory action of AZD. SIRT1, a NAD-dependent deacetylase, functions as an intracellular regulatory protein and is known to regulate inflammation in various diseases [35,36]. Our results showed an increase in SIRT-1 expression after B(a)P treatment, which increased still further upon treatment with AZD (Figure 11D). SIRT1 could have been activated as a cellular response to the inflammation caused by NF-κB p65 release. AZD increased SIRT1 expression and regulated the inflammatory response.

**Figure 12:**
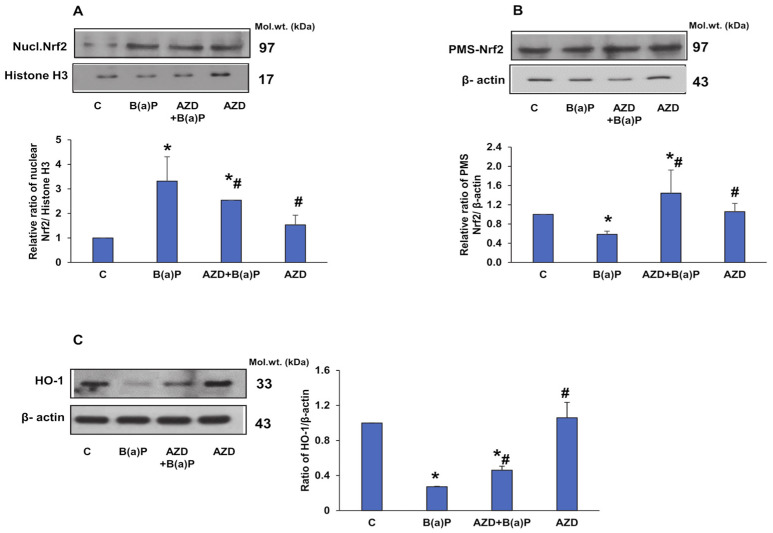
Expression of regulatory markers of cellular defense induced by B(a)P. Sub-cellular fractions (nuclear and post-mitochondria) from HepG2 cells treated with B(a)P and/or AZD resolved using 12% SDS-PAGE, transferred via Western blotting, and immunoblotted using the protein-specific antibodies against nuclear Nrf2 (**A**), cytosolic Nrf2 (**B**), and HO-1 (**C**). Immunoreactive proteins were visualized via enhanced chemiluminescence using Sapphire Biomolecular Imager (Azure biosystems, Dublin, CA, USA) or by developing them on X-ray films. Beta-actin and Histone H3 were used as loading controls for total/post-mitochondrial and nuclear fractions, respectively. Image Studio Lite Ver.5.2 (LI-COR Biosciences, Lincoln, NE, USA) was used for the densitometric analysis of the protein bands and these are represented as ratios relative to their appropriate loading controls and as histograms. A typical representation of three replicate experiments is shown. Asterisks represent significant differences and are fixed at *p* ≤ 0.05 (* represents significant differences with respect to control cells whereas # represents significant differences with respect to B(a)P-treated cells). Molecular weights are expressed in kDa.

Nrf2 (nuclear factor erythroid 2-related factor) controls the cellular defense mechanism by regulating the redox balance and the xenobiotic detoxification system [37]. It is known to accumulate in the nucleus in response to oxidative stress and activates the antioxidant response element-dependent target genes [38]. Our study also confirmed this finding. Increased translocation of Nrf2 into the nucleus was observed upon treatment with B(a)P (Figure 12A,B). AZD treatment attenuated the translocation and increased the cytosolic levels of Nrf2. However, the expression of HO-1, another redox-sensitive marker, was reduced after B(a)P treatment (Figure 12C). AZD slightly increased the HO-1 level, though it was still significantly lower than the control level.

**Figure 13:**
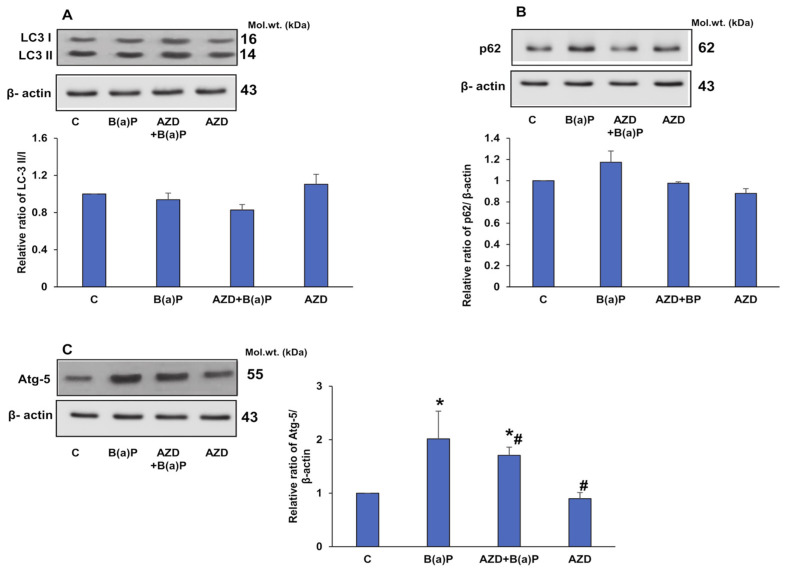
Expression of autophagy markers induced by B(a)P. Total cell extracts from HepG2 cells treated with B(a)P and/or AZD resolved using 12% SDS-PAGE, transferred via Western blotting, and immunoblotted with antibodies against LC3 (**A**), p62 (**B**), and Atg-5 (**C**). Immunoreactive proteins visualized via enhanced chemiluminescence using Sapphire Biomolecular Imager (Azure biosystems, Dublin, CA, USA) or by developing them on X-ray films. The loading control was beta-actin. Image Studio Lite Ver.5.2 (LI-COR Biosciences, Lincoln, NE, USA) was used for densitometric analysis and the results are expressed as ratios relative to their appropriate loading proteins and represented as histograms. A typical representation of three replicate experiments is shown. Asterisks represent significant differences and are fixed at *p* ≤ 0.05 (* represents significant differences with respect to control cells whereas # represents significant differences with respect to B(a)P-treated cells). Molecular weights are expressed in kDa.

To further check if the cellular defense mechanism activated the autophagic machinery, we investigated the expression of autophagy markers. A two-fold increase in Atg-5 protein expression and a moderate increase in p62 protein expression was observed after B(a)P treatment (Figure 13A,B), which was reduced upon treatment with AZD. The expression of LC3 proteins, however, remained unaltered. The results suggest a cell survival attempt by the cells but a failure to activate the autophagic cascade, thus resulting in increased apoptotic cell death.

### 3.7. B(a)P-Induced Alterations in the Cell-Signaling Markers

**Figure 14:**
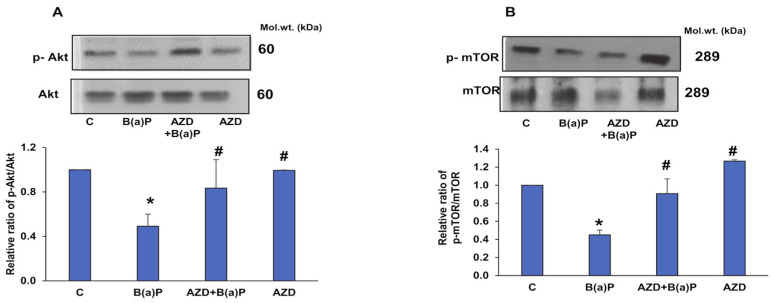
B(a)P-induced attenuation in cell signaling protein expression. Cell homogenates from B(a)P- and/or AZD- treated HepG2 cells resolved using 12% SDS-PAGE, transferred via Western blotting, and immunoblotted using the protein-specific antibodies against Akt/p-Akt (**A**) and mTOR/p-mTOR (**B**). Immunoreactive proteins were visualized via enhanced chemiluminescence using Sapphire Biomolecular Imager (Azure biosystems, Dublin, CA, USA) or by developing them on X-ray films. Bar diagrams represent the ratios of the phosphorylated proteins relative to the respective total proteins. A typical representation of three replicate experiments is shown. Asterisks represent significant differences and are fixed at *p* ≤ 0.05 (* represents significant differences with respect to control cells whereas # represents significant differences with respect to B(a)P-treated cells). Molecular weights are expressed in kDa.

To further explore the cross-talk between cell survival, oxidative stress, and apoptotic cascades, we investigated the expression of some critical cell-signaling markers. Akt is an important regulator of cell survival and apoptosis. As shown in Figure 14A, we observed a decreased expression of Akt after B(a)P treatment, compared to that of control cells, which increased significantly after AZD treatment. AZD alone did not show any alteration compared to the control. Concomitantly, we also observed a decreased phosphorylation of mTOR (mammalian target of rapamycin) after B(a)P treatment, which was alleviated in the presence of AZD (Figure 14B). mTOR is known to be a crucial metabolic and cell growth regulator and is modulated in response to oxidative stress. AZD treatment alone showed a slight increase in expression compared to that in control cells.

## 4. Discussion

B(a)P, a product of cigarette smoke, grilled or burnt food, and various other sources, has been known to play crucial role in the pathogenesis of various cancers, atherosclerosis, and neurodegenerative diseases in humans [2,14]. B(a)P is metabolized to generate 7,8-diol-9,10-epoxide (BPDE), which forms adducts with DNA, causing DNA damage, ROS production, apoptosis, inflammation, and oxidative and metabolic stress, leading to cancer [3,6].

The use of natural, herbal, or dietary supplements like polyphenols has become a promising strategy to protect against B(a)P-induced damage [6,14]. Researchers have been trying to study the effects of various biologically active anti-oxidants to assess their ability to withstand the toxicity/carcinogenicity caused by B(a)P in vitro and in vivo [13,14,15,16]. Azadirachtin (AZD), a limonoid obtained from the tropical neem tree (*Azadirachta indica*) is a well-known anti-oxidant, anti-inflammatory, and anti-cancer agent [17,18,19]. These effects have been demonstrated in vitro in carcinogenic cell lines as well as in animal models [22]. Our previous studies have also reported that AZD treatment could enhance cellular survival under conditions of extreme inflammatory and oxidative stress through an interconnecting network of cell-signaling proteins [20,21]. Our present study focuses on the mitigation of oxidative, inflammatory, and metabolic stress by AZD in B(a)P-treated HepG2 cells. We observed a marked reduction in B(a)P-induced oxidative stress and apoptosis, an improvement in anti-oxidant status, DNA damage, redox homeostasis, and mitochondrial bioenergetics caused by AZD.

Many studies have reported B(a)P-stimulated oxidative stress caused by intracellular ROS production, lipid peroxidation, and alterations in anti-oxidant enzymes in vitro and in vivo [3,6,39,40]. Our findings also demonstrated increased oxidative/nitrosative stress in HepG2 cells treated with 25 µM B(a)P for 24 h and its attenuation with AZD. We observed a significant increase in total ROS and mitochondrial ROS production as well as membrane-bound NADPH oxidase-dependent ROS production. Treatment with AZD significantly reduced ROS production. A reduction in mitochondrial ROS with AZD treatment was confirmed using a ROS-sensitive, mitochondrial-specific fluorogenic probe, CM-H2XRos (Mito Tracker^®^ Red), which specifically measures the intracellular production of hydrogen peroxide. Studies have shown the increased cellular content of MDA after treatment with B(a)P, indicating increased oxidative stress [6,39,41,42,43]. Our study also confirmed increased MDA levels after B(a)P treatment, which were alleviated in the presence of AZD. We also observed increased NO production after B(a)P treatment, which was attenuated in the presence of AZD. B(a)P-induced iNOS induction resulting in increased oxidative stress and apoptosis was confirmed in an earlier study [44].

Numerous studies have proposed B(a)P-induced cellular apoptosis as a series of multiple pathophysiological events including the activation of caspases, PARP, the elevation of pro-apoptotic Bax, a decrease in anti-apoptotic Bcl-2 protein, and the release of cytochrome c in vitro and in vivo [10,45,46]. Dietary polyphenols and other anti-oxidants like melatonin, resveratrol, quercetin, catechins, and related compounds have been shown to reverse and regulate B(a)P-induced apoptosis [10,47]. Our findings also demonstrated increased apoptosis and the activation of caspase-8 and -9, leading to the cleavage of caspase-3 and PARP. In addition, we also observed an increased expression of Bax, accompanied by a decreased expression of Bcl-2 and the release of cytochrome c to the cytosol after B(a)P treatment, indicating B(a)P-induced intrinsic and extrinsic apoptosis. All these changes were reversed by AZD.

Since our findings confirmed that mitochondrial ROS production and the mitochondrially mediated intrinsic apoptotic pathway are involved in B(a)P-induced toxicity, we went further to study the mitochondrial membrane potential (MMP) and activities of the respiratory enzyme complexes and their bioenergetics. Consistent with other studies [3,10,39,40,48,49], we also observed a three- to four-fold loss of membrane potential after B(a)P treatment, followed by the release of cytochrome c into the cytosol. AZD treatment abrogated the loss and brought the values close to control levels. In addition, we observed a recovery of the decreased activities of the mitochondrial respiratory complexes and reduced ATP production after AZD treatment. Aconitase, a ROS-sensitive Krebs’ cycle enzyme, also showed partial recovery after AZD treatment. This was confirmed by immunoblotting. Studies have suggested that alterations in the tricarboxylic acid cycle likely involve the dysfunction of mitochondrial Complex II [50].

The first physiological line of defense against oxidative stress comprises anti-oxidant enzymes including SOD, catalase, GSH, and GSH-metabolizing enzymes [39]. B(a)P administration has been shown to suppress SOD activity [6,39,40,44], indicating increased oxidative stress. Our study also showed decreased SOD activity and increased catalase activity in the cells treated with B(a)P, which were regulated after AZD treatment. AZD treatment also augmented the B(a)P-decreased expression of the anti-oxidant marker protein, hemeoxygenase-1 (HO-1). This decrease in HO-1 in response to B(a)P was seen in an earlier study [51]. These findings clearly indicate the anti-oxidant property of AZD.

However, we found an increase in the level of GSH and its metabolizing enzymes, GST and GSH-Px, after B(a)P treatment. Studies have shown that GSH levels and GST activity are highest at 24 h of B(a)P treatment, and then decrease until 72 h, whereas GSH-Px activity continues increasing until 72 h [28,34], indicating glutathione conjugation as an important pathway for B(a)P detoxification. Confirming this, our findings also demonstrated increased GSH levels, in addition to increased GST and GSH-Px activities after treatment with B(a)P for 24 h in both the mitochondrial as well as the post-mitochondrial fractions, which were alleviated after AZD treatment. However, we observed a decrease in GSH reductase activity, indicating the reduced conversion of the oxidized glutathione into reduced glutathione, causing a disturbance in cellular functions and redox homeostasis. This was again modulated by AZD, which again reaffirms the anti-oxidant potential of AZD.

Nrf2 (nuclear factor erythroid 2-related factor), a transcription factor and cellular defense regulator, activates the transcription of anti-oxidative enzymes, and anti-inflammatory and detoxification proteins, and can minimize protein and DNA damage by degrading bioactive molecules [27,37,38]. Increased oxidative stress has also been shown to elevate Nrf2 levels, leading to the significant activation of GST in vitro and in vivo [39,52]. Nrf2 thus plays a central role in balancing cell homeostasis by balancing the production and scavenging of ROS [37]. Our study demonstrated that AZD treatment attenuated the B(a)P-induced activation of Nrf2 and the induction of the GST detoxification enzyme.

NF–κB, an inducible transcription factor, plays a significant role in regulating cell inflammation, proliferation, and apoptosis, and resides in an inactive form in the cytoplasm complexed with IκB. Upon activation, IκB undergoes phosphorylation and NF–κB is translocated to the nucleus [41]. We also observed increased translocation of NF–κB to the nucleus upon B(a)P treatment which was attenuated in the presence of AZD. In addition, AZD treatment reduced the expression of cytosolic IκB, confirming the decreased translocation of NF–κB. As shown earlier [3], our study also confirmed the induction of NF–κB due to the upregulation of Bax, downregulation of Bcl-2, and release of cytochrome c into the cytosol after B(a)P treatment, which were attenuated in the presence of AZD.

SIRT1 (sirtuin 1), an intracellular NAD-dependent deacetylase, is known to deacetylate target proteins and modulate the inflammatory response manifested by inflammatory cytokines [35,36]. In our study, we observed the activation of SIRT1 upon treatment with B(a)P. This could be the cell’s survival response to B(a)P-induced NFκB activation. Studies have also reported that SIRT1 regulates apoptosis and autophagic pathways [10]. As demonstrated in this study, our study also showed a B(a)P-induced increase in the expression of the Atg-5 and p62 proteins, which was reduced upon treatment with AZD. However, no alterations were observed in the LC3 proteins, indicating failure to activate the autophagic machinery, resulting in apoptotic cell death. AZD treatment attenuated the expression of Atg-5 and p62 proteins with no alterations in LC3 proteins, again confirming that the autophagic machinery was not involved. Researchers have reported a cross-talk between SIRT1 and Nrf2 associated with cyclin B1 expression [37]. Our study confirmed this observation via the B(a)P-induced activation of SIRT1, Nrf2, and cyclin B1 expression.

Studies have also shown the regulation of autophagy through the AMPK/mTOR pathway [53]. Our results showed a decreased expression of mTOR after B(a)P treatment, which recovered slightly after AZD treatment. B(a)P has been shown to markedly reduce the protein expression of p-Akt and consequently induce ‘pyroptic’ cell death, characterized by the release of inflammatory factors, including NO [54]. Our study confirmed the repression of p-Akt expression and an increase in NO levels, which were recovered after AZD treatment.

B(a)P is metabolized by cytochrome P450 (CYP 450) enzymes, mainly the CYP 1A enzymes, generating epoxides, which can cause DNA damage and initiate carcinogenesis [43,48,55]. Our present study also showed increased activities of CYP 450 1A1 and 1A2 after B(a)P treatment, which were alleviated in the presence of AZD. This was further confirmed via immunoblotting.

Since B(a)P metabolites produced by CYP 1A biotransformation can cause DNA damage, we examined DNA fragmentation using electrophoresis and comet assay and observed increased DNA strand breaks after B(a)P treatment, which were ameliorated after AZD treatment. We observed a pronounced B(a)P-induced cleavage of the DNA repair enzyme, PARP, which again was reduced in the presence of AZD. This could be due to reduced DNA breakdown after AZD treatment. Oxidative DNA damage also results in the accumulation of p53, which in turn upregulates the cellular p21 protein [43,56]. Our study confirmed this finding. We observed an increased expression of p53 and p21 proteins after B(a)P treatment, which reduced significantly after AZD treatment. This accumulation of p53 could be a downstream response to DNA damage, which in turn could cause cell cycle arrest (indicated by p21 activation) or apoptosis. The p53 protein is also known to regulate apoptosis by transcriptionally inducing the expression of the pro-apoptotic Bax protein [43,57,58].

To test the role of p21 in cell cycle progression, we determined the effects of B(a)P and/or AZD treatment on cell cycle distribution. We observed a significant increase in the percentage of cells in the S and G2/M phase and a concomitant reduction in the G0/G1 phase after B(a)P treatment for 24 h, with a partial recovery after AZD treatment. Consistent with our results, other researchers found that B(a)P caused cell cycle arrest in the S- and G2/M phases in MCF-7 and other cell lines, in spite of the increased accumulation of p53 and p21 proteins [59,60,61,62].

Cell cycle progression is known to be regulated by cyclin/Cdk interactions [63]. We observed a significant increase in the expressions of cyclin B1, cyclin D1, Cdk2, and Cdk4 after B(a)P treatment, which was attenuated in the presence of AZD. Consistent with our findings, another group of researchers have shown increased levels of both Cdk1 and cyclin B1 accompanied by G2 arrest after BPDE treatment, which could be due to decreased levels of Cdc 25B and Cdc 25C proteins [57]. The overexpression of cyclin D1, a significant regulator of the G1/S phase, along with the increased synthesis of the Cdk4 protein, was shown to be a critical requirement in B(a)P-induced cell cycle progression [64,65]. A recent review suggested that abnormalities in cyclin D and/or the overexpression of Cdk4/6 can promote tumorigenesis and are deemed critical therapeutic targets for multiple cancers [66,67]. In our study, AZD attenuated the cell cycle regulatory proteins cyclin B1, D1, and Cdks 2 and 4, suggesting its potential for use as a promising anti-cancer therapeutic in combination with anti-cancer drugs.

## 5. Conclusions

The use of natural products, or dietary supplements like polyphenols, has become a promising strategy as a defense against the damaging and detrimental effects of various carcinogens, including B(a)P. Numerous studies have shown that biologically active compounds and polyphenols have the ability to reduce oxidative stress, DNA damage, and carcinogenesis induced by B(a)P. Studies have demonstrated the beneficial effects of Azadirachtin (AZD), a limonoid extracted from the tropical neem tree (*Azadirachta indica*) as an anti-oxidant, anti-inflammatory, and anti-cancer agent. To the best of our knowledge, this is the first comprehensive study of its kind showing the beneficial effects of AZD on B(a)P-induced oxidative stress and apoptosis, anti-oxidant status, DNA damage, redox homeostasis, and mitochondrial bioenergetics. In addition, alterations in inflammatory, cell-signaling, and cell cycle regulatory markers were also observed. These anti-oxidant, anti-inflammatory, and regulatory effects of AZD on cell cycle markers can be used as a prospective strategy in combination therapy for the treatment of different malignancies.

## 6. Limitations

In our present study, we studied the beneficial effects of AZD on HepG2 cells treated with only 25 µM B(a)P for 24 h. We need to extrapolate this study to check the effects of AZD on the long-term treatment of B(a)P to mimic normal human environmental exposures, which is part of our ongoing studies. We also need to include in vivo studies to confirm the results from our present study.

## Data Availability

All data are provided in the manuscript.

## References

[1] Chhikara B.S., Parang K. (2023). Global Cancer Statistics 2022: The Trends Projection Analysis. Chem. Biol. Lett..

[2] U.S. EPA (2017). IRIS Toxicological Review of Benzo[A]Pyrene (Final Report).

[3] Liu B.-Y., Chiou J.-Z., Huang K.-M., Chen T.-Y., Hwang D.-F. (2022). Effects of Taurine against Benzo[α]Pyrene-Induced Cell Cycle Arrest and Reactive Oxygen Species-Mediated Nuclear Factor-Kappa B Apoptosis via Reduction of Mitochondrial Stress in A549 Cells. Chin. J. Physiol..

[4] Nemmar A., Raza H., Subramaniyan D., Yasin J., John A., Ali B.H., Kazzam E.E. (2013). Short-Term Systemic Effects of Nose-Only Cigarette Smoke Exposure in Mice: Role of Oxidative Stress. Cell. Physiol. Biochem..

[5] Raza H., John A., Nemmar A. (2013). Short-Term Effects of Nose-Only Cigarette Smoke Exposure on Glutathione Redox Homeostasis, Cytochrome P450 1A1/2 and Respiratory Enzyme Activities in Mice Tissues. Cell. Physiol. Biochem..

[6] Wang C., Zhao F., Bai Y., Li C., Xu X., Kristiansen K., Zhou G. (2022). Proteomic Analysis of the Protective Effect of Eriodictyol on Benzo(a)Pyrene-Induced Caco-2 Cytotoxicity. Front. Nutr..

[7] IARC Working Group on the Evaluation of Carcinogenic Risks to Humans (2010). Some Non-Heterocyclic Polycyclic Aromatic Hydrocarbons and Some Related Exposures. IARC Monogr. Eval. Carcinog. Risks Hum..

[8] He J., Pang Q., Huang C., Xie J., Hu J., Wang L., Wang C., Meng L., Fan R. (2022). Environmental Dose of 16 Priority-Controlled PAHs Mixture Induce Damages of Vascular Endothelial Cells Involved in Oxidative Stress and Inflammation. Toxicol. Vitr..

[9] Yang Y., Jin M., Meng Y., Dai Y., Chen S., Zhou Y., Li Y., Tang L. (2023). Involvement and Targeted Intervention of Benzo(a)Pyrene-Regulated Apoptosis Related Proteome Modification and Muti-Drug Resistance in Hepatocellular Carcinoma. Cell Death Dis..

[10] Barangi S., Mehri S., Moosavi Z., Hayesd A.W., Reiter R.J., Cardinali D.P., Karimi G. (2020). Melatonin Inhibits Benzo(a)Pyrene-Induced Apoptosis through Activation of the Mir-34a/Sirt1/Autophagy Pathway in Mouse Liver. Ecotoxicol. Environ. Saf..

[11] Ba Q., Li J., Huang C., Qiu H., Li J., Chu R., Zhang W., Xie D., Wu Y., Wang H. (2015). Effects of Benzo[a]Pyrene Exposure on Human Hepatocellular Carcinoma Cell Angiogenesis, Metastasis, and NF-ΚB Signaling. Environ. Health Perspect..

[12] Bucher S., Tête A., Podechard N., Liamin M., Le Guillou D., Chevanne M., Coulouarn C., Imran M., Gallais I., Fernier M. (2018). Co-Exposure to Benzo[a]Pyrene and Ethanol Induces a Pathological Progression of Liver Steatosis In Vitro and In Vivo. Sci. Rep..

[13] Lee S.-C., Jee S.-C., Kim M., Kim S., Shin M.K., Kim Y., Sung J.-S. (2021). Curcumin Suppresses the Lipid Accumulation and Oxidative Stress Induced by Benzo[a]Pyrene Toxicity in HepG2 Cells. Antioxidants.

[14] Bukowska B., Duchnowicz P. (2022). Molecular Mechanisms of Action of Selected Substances Involved in the Reduction of Benzo[a]Pyrene-Induced Oxidative Stress. Molecules.

[15] Zhu W., Cromie M.M., Cai Q., Lv T., Singh K., Gao W. (2014). Curcumin and Vitamin E Protect against Adverse Effects of Benzo[a]Pyrene in Lung Epithelial Cells. PLoS ONE.

[16] Kumar M., Sharma V.L., Sehgal A., Jain M. (2012). Protective Effects of Green and White Tea Against Benzo(a)Pyrene Induced Oxidative Stress and DNA Damage in Murine Model. Nutr. Cancer.

[17] Schumacher M., Cerella C., Reuter S., Dicato M., Diederich M. (2011). Anti-Inflammatory, pro-Apoptotic, and Anti-Proliferative Effects of a Methanolic Neem (Azadirachta Indica) Leaf Extract Are Mediated via Modulation of the Nuclear Factor-ΚB Pathway. Genes Nutr..

[18] Fernandes S.R., Barreiros L., Oliveira R.F., Cruz A., Prudêncio C., Oliveira A.I., Pinho C., Santos N., Morgado J. (2019). Chemistry, Bioactivities, Extraction and Analysis of Azadirachtin: State-of-the-Art. Fitoterapia.

[19] Dubey R., Patil K., Dantu S.C., Sardesai D.M., Bhatia P., Malik N., Acharya J.D., Sarkar S., Ghosh S., Chakrabarti R. (2019). Azadirachtin Inhibits Amyloid Formation, Disaggregates Pre-Formed Fibrils and Protects Pancreatic β-Cells from Human Islet Amyloid Polypeptide/Amylin-Induced Cytotoxicity. Biochem. J..

[20] John A., Raza H. (2021). Azadirachtin Attenuates Lipopolysaccharide-Induced ROS Production, DNA Damage, and Apoptosis by Regulating JNK/Akt and AMPK/MTOR-Dependent Pathways in Rin-5F Pancreatic Beta Cells. Biomedicines.

[21] John A., Raza H. (2022). Alterations in Inflammatory Cytokines and Redox Homeostasis in LPS-Induced Pancreatic Beta-Cell Toxicity and Mitochondrial Stress: Protection by Azadirachtin. Front. Cell. Dev. Biol..

[22] Siddavaram N., Palrasu M., Bishayee A. (2023). Limonoids from Neem (*Azadirachta indica* A. Juss.) Are Potential Anticancer Drug Candidates. Med. Res. Rev..

[23] Gangar S.C., Koul A. (2007). Azadirachta Indica Leaf Extract Modulates Initiation Phase of Murine Forestomach Tumorigenesis. Indian J. Biochem. Biophys..

[24] Gangar S.C., Koul A. (2008). Azadirachta Indica Modulates Carcinogen Biotransformation and Reduced Glutathione at Peri-Initiation Phase of Benzo(a)Pyrene Induced Murine Forestomach Tumorigenesis. Phytother. Res..

[25] Gangar S.C., Koul A. (2008). Histochemical, Ultrastructural, and Biochemical Evidences for Azadirachta Indica- Induced Apoptosis in Benzo (a) Pyrene- Induced Murine Forestomach Tumors. J. Environ. Pathol. Toxicol. Oncol..

[26] Gangar S.C., Sandhir R., Rai D.V., Koul A. (2006). Modulatory Effects of Azadirachta Indica on Benzo(a)Pyrene-Induced Forestomach Tumorigenesis in Mice. World J. Gastroenterol..

[27] van Delft J.H.M., Mathijs K., Staal Y.C.M., van Herwijnen M.H.M., Brauers K.J.J., Boorsma A., Kleinjans J.C.S. (2010). Time Series Analysis of Benzo[A]Pyrene-Induced Transcriptome Changes Suggests That a Network of Transcription Factors Regulates the Effects on Functional Gene Sets. Toxicol. Sci..

[28] Lin T., Yang M.S. (2007). Benzo[a]Pyrene-Induced Elevation of GSH Level Protects against Oxidative Stress and Enhances Xenobiotic Detoxification in Human HepG2 Cells. Toxicology.

[29] Alnahdi A., John A., Raza H. (2020). Mitigation of Glucolipotoxicity-Induced Apoptosis, Mitochondrial Dysfunction, and Metabolic Stress by N-Acetyl Cysteine in Pancreatic β-Cells. Biomolecules.

[30] Raza H., John A. (2015). Differential Cytotoxicity of Acetaminophen in Mouse Macrophage J774.2 and Human Hepatoma HepG2 Cells: Protection by Diallyl Sulfide. PLoS ONE.

[31] Alnahdi A., John A., Raza H. (2019). N-Acetyl Cysteine Attenuates Oxidative Stress and Glutathione-Dependent Redox Imbalance Caused by High Glucose/High Palmitic Acid Treatment in Pancreatic Rin-5F Cells. PLoS ONE.

[32] John A., Howarth F.C., Raza H. (2022). Exercise Alleviates Diabetic Complications by Inhibiting Oxidative Stress-Mediated Signaling Cascade and Mitochondrial Metabolic Stress in GK Diabetic Rat Tissues. Front. Physiol..

[33] Birch-Machin M.A., Turnbull D.M. (2001). Assaying Mitochondrial Respiratory Complex Activity in Mitochondria Isolated from Human Cells and Tissues. Methods Cell Biol..

[34] Deng C., Dang F., Gao J., Zhao H., Qi S., Gao M. (2018). Acute Benzo[a]Pyrene Treatment Causes Different Antioxidant Response and DNA Damage in Liver, Lung, Brain, Stomach and Kidney. Heliyon.

[35] Lei Y., Zhu Y., Mallah M.A., Lu P., Yang L., He X., Shang P., Chen Y., Zhou X., Feng F. (2023). The Activation of SIRT1 Ameliorates BPDE-Induced Inflammatory Damage in BEAS-2B Cells via HMGB1/TLR4/NF-ΚB Pathway. Environ. Toxicol..

[36] Lu J., Zhang M., Huang Z., Sun S., Zhang Y., Zhang L., Peng L., Ma A., Ji P., Dai J. (2015). SIRT1 in B[a]P-Induced Lung Tumorigenesis. Oncotarget.

[37] Yagishita Y., Chartoumpekis D.V., Kensler T.W., Wakabayashi N. (2023). NRF2 and the Moirai: Life and Death Decisions on Cell Fates. Antioxid. Redox Signal..

[38] Nguyen P.M., Park M.S., Chow M., Chang J.H., Wrischnik L., Chan W.K. (2010). Benzo[a]Pyrene Increases the Nrf2 Content by Downregulating the Keap1 Message. Toxicol. Sci..

[39] Washimkar K.R., Tomar M.S., Ishteyaque S., Kumar A., Shrivastava A., Mugale M.N. (2023). Benzo[a]Pyrene Treatment Modulates Nrf2/Keap1 Axis and Changes the Metabolic Profile in Rat Lung Cancer. Chem. Biol. Interact..

[40] Lyu Y., Yang J., Cheng L., Li Z., Zheng J. (2023). Benzo(a)Pyrene-Induced Mitochondrial Respiration and Glycolysis Disturbance in Human Neuroblastoma Cells. J. Toxicol. Sci..

[41] Ji K., Xing C., Jiang F., Wang X., Guo H., Nan J., Qian L., Yang P., Lin J., Li M. (2013). Benzo[a]Pyrene Induces Oxidative Stress and Endothelial Progenitor Cell Dysfunction via the Activation of the NF-ΚB Pathway. Int. J. Mol. Med..

[42] Nithya G., Santhanasabapathy R., Vanitha M.K., Anandakumar P., Sakthisekaran D. (2023). Antioxidant, Antiproliferative, and Apoptotic Activity of Thymoquinone against Benzo(a)Pyrene-Induced Experimental Lung Cancer. J. Biochem. Mol. Toxicol..

[43] Banerjee B., Chakraborty S., Ghosh D., Raha S., Sen P.C., Jana K. (2016). Benzo(a)Pyrene Induced P53 Mediated Male Germ Cell Apoptosis: Synergistic Protective Effects of Curcumin and Resveratrol. Front. Pharmacol..

[44] Dutta K., Ghosh D., Nazmi A., Kumawat K.L., Basu A. (2010). A Common Carcinogen Benzo[a]Pyrene Causes Neuronal Death in Mouse via Microglial Activation. PLoS ONE.

[45] Holme J.A., Gorria M., Arlt V.M., Øvrebø S., Solhaug A., Tekpli X., Landvik N.E., Huc L., Fardel O., Lagadic-Gossmann D. (2007). Different Mechanisms Involved in Apoptosis Following Exposure to Benzo[a]Pyrene in F258 and Hepa1c1c7 Cells. Chem. Biol. Interact..

[46] Kim J.Y., Chung J.-Y., Park J.-E., Lee S.G., Kim Y.-J., Cha M.-S., Han M.S., Lee H.-J., Yoo Y.H., Kim J.-M. (2007). Benzo[a]Pyrene Induces Apoptosis in RL95-2 Human Endometrial Cancer Cells by Cytochrome P450 1A1 Activation. Endocrinology.

[47] Khattab S.A., Hussien W.F., Raafat N., Ahmed Alaa El-Din E. (2021). Effects of Catechin Hydrate in Benzo[a]Pyrene-Induced Lung Toxicity: Roles of Oxidative Stress, Apoptosis, and DNA Damage. Toxicol. Mech. Methods.

[48] Chen S. (2003). The Role of the Ah Receptor and P38 in Benzo[a]Pyrene-7,8-Dihydrodiol and Benzo[a]Pyrene-7,8-Dihydrodiol-9,10-Epoxide-Induced Apoptosis. J. Biol. Chem..

[49] Nie J.-S., Zhang H.-M., Zhao J., Liu H.-J., Niu Q. (2014). Involvement of Mitochondrial Pathway in Benzo[a]Pyrene-Induced Neuron Apoptosis. Hum. Exp. Toxicol..

[50] Hardonnière K., Saunier E., Lemarié A., Fernier M., Gallais I., Héliès-Toussaint C., Mograbi B., Antonio S., Bénit P., Rustin P. (2016). The Environmental Carcinogen Benzo[a]Pyrene Induces a Warburg-like Metabolic Reprogramming Dependent on NHE1 and Associated with Cell Survival. Sci. Rep..

[51] Lou Y., Guo Z., Zhu Y., Kong M., Zhang R., Lu L., Wu F., Liu Z., Wu J. (2019). Houttuynia Cordata Thunb. and Its Bioactive Compound 2-Undecanone Significantly Suppress Benzo(a)Pyrene-Induced Lung Tumorigenesis by Activating the Nrf2-HO-1/NQO-1 Signaling Pathway. J. Exp. Clin. Cancer Res..

[52] Jee S.-C., Kim M., Sung J.-S. (2020). Modulatory Effects of Silymarin on Benzo[a]Pyrene-Induced Hepatotoxicity. Int. J. Mol. Sci..

[53] Tasdemir E., Maiuri M., Galluzzi L., Vitale I., Djavaheri-Mergny M., D’amelio M., Criollo A., Morselli E., Zhu C., Harper F. (2008). Regulation of autophagy by cytoplasmic p53. Nat. Cell Biol..

[54] Li Q., Gao C., Deng H., Song Q., Yuan L. (2019). Benzo(a)pyrene induces pyroptic and autophagic death through inhibiting PI3K/Akt signaling pathway in HL-7702 human normal liver cells. J. Toxicol. Sci..

[55] Genies C., Maître A., Lefèbvre E., Jullien A., Chopard-Lallier M., Douki T. (2013). The Extreme Variety of Genotoxic Response to Benzo[a]Pyrene in Three Different Human Cell Lines from Three Different Organs. PLoS ONE.

[56] Park S.-Y., Lee S.-M., Ye S.-K., Yoon S.-H., Chung M.-H., Choi J. (2006). Benzo[a]Pyrene-Induced DNA Damage and P53 Modulation in Human Hepatoma HepG2 Cells for the Identification of Potential Biomarkers for PAH Monitoring and Risk Assessment. Toxicol. Lett..

[57] Xiao H., Singh S.V. (2007). P53 Regulates Cellular Responses to Environmental Carcinogen Benzo[a]Pyrene-7,8-Diol-9,10-Epoxide in Human Lung Cancer Cells. Cell Cycle.

[58] Asweto C.O., Wu J., Hu H., Feng L., Yang X., Duan J., Sun Z. (2017). Combined Effect of Silica Nanoparticles and Benzo[a]Pyrene on Cell Cycle Arrest Induction and Apoptosis in Human Umbilical Vein Endothelial Cells. Int. J. Environ. Res. Public Health.

[59] Hamouchene H., Arlt V.M., Giddings I., Phillips D.H. (2011). Influence of Cell Cycle on Responses of MCF-7 Cells to Benzo[a]Pyrene. BMC Genom..

[60] Jeffy B.D., Chen E.J., Gudas J.M., Romagnolo D.F. (2000). Disruption of Cell Cycle Kinetics by Benzo[a]Pyrene: Inverse Expression Patterns of BRCA-1 and P53 in MCF-7 Cells Arrested in S and G2. Neoplasia.

[61] Gao M., Zheng A., Chen L., Dang F., Liu X., Gao J. (2022). Benzo(a)Pyrene Affects Proliferation with Reference to Metabolic Genes and ROS/HIF-1α/HO-1 Signaling in A549 and MCF-7 Cancer Cells. Drug Chem. Toxicol..

[62] Khan Q.A., Dipple A., Anderson L.M. (2002). Protease Inhibitor-Induced Stabilization of P21(Waf1/Cip1) and Cell-Cycle Arrest in Chemical Carcinogen-Exposed Mammary and Lung Cells. Mol. Carcinog..

[63] Xu H., Yi T., Liu M., Gao R., Liu X., He J., Ding Y., Geng Y., Mu X., Wang Y. (2023). Exposure to Benzo(a)Pyrene Promotes Proliferation and Inhibits Differentiation of Stromal Cells in Mice during Decidualization. Ecotoxicol. Environ. Saf..

[64] Du H.J., Tang N., Liu B.C., You B.R., Shen F.H., Ye M., Gao A., Huang C.S. (2006). Benzo[a]Pyrene-Induced Cell Cycle Progression Is through ERKs/Cyclin D1 Pathway and Requires the Activation of JNKs and P38 Mapk in Human Diploid Lung Fibroblasts. Mol. Cell Biochem..

[65] Ding J., Ning B., Gong W., Wen W., Wu K., Liang J., He G., Huang S., Sun W., Han T. (2009). Cyclin D1 Induction by Benzo[a]Pyrene-7,8-Diol-9,10-Epoxide via the Phosphatidylinositol 3-Kinase/Akt/MAPK- and P70s6k-Dependent Pathway Promotes Cell Transformation and Tumorigenesis. J. Biol. Chem..

[66] Qi J., Ouyang Z. (2022). Targeting CDK4/6 for Anticancer Therapy. Biomedicines.

[67] Du Q., Guo X., Wang M., Li Y., Sun X., Li Q. (2020). The Application and Prospect of CDK4/6 Inhibitors in Malignant Solid Tumors. J. Hematol. Oncol..

